# Measuring resilience and stress during pregnancy and its relation to vulnerability and pregnancy outcomes in a nulliparous cohort study

**DOI:** 10.1186/s12884-023-05692-5

**Published:** 2023-05-29

**Authors:** Anic C. Alves, Renato T. Souza, Jussara Mayrink, Rafael B. Galvao, Maria L. Costa, Francisco E. Feitosa, Edilberto A. Rocha Filho, Débora F. Leite, Ricardo P. Tedesco, Danielly S. Santana, Karayna G. Fernandes, Maria J. Miele, Joao P. Souza, Jose G. Cecatti, MAESI Study Group, Daisy Lucena, Denise Ellen F. Cordeiro, Danilo Anacleto, Lívia C. Nascimento, Mariana B. Rogerio, Francisco Barbosa Junior

**Affiliations:** 1 Department of Obstetrics and Gynecology, School of Medical Sciences, University of Campinas (UNICAMP), 101 Alexander Fleming, Cidade Universitária, Campinas, SP Brazil; 2 Federal University of Ceará, Fortaleza, CE Brazil; 3 Department of Gynecology and Obstetrics, Medical Sciences School, Federal University of Pernambuco, Recife, PE Brazil; 4 Department of Obstetrics and Gynecology, Jundiaí Medical School, Jundiaí, SP Brazil; 5 Department of Social Medicine, Ribeirão Preto Medical School, University of Sao Paulo, Ribeirão Preto, SP Brazil

**Keywords:** Resilience, Stress, Pregnancy, Vulnerability, Validation, Scale

## Abstract

**Background:**

Resilience reflects coping with pregnancy-specific stress, including physiological adaptations of the maternal organism or factors arising from the socioeconomic context, such as low income, domestic violence, drug and alcohol use, lack of a support network and other vulnerability characteristics. Resilience is a dynamic characteristic that should be comparatively evaluated within a specific context; its association with perceived stress and social vulnerability during pregnancy is still not fully understood. This study aimed at exploring maternal resilience, perceived stress and social vulnerability during pregnancy and its associated factors and outcomes.

**Methods:**

Prospective multicenter cohort study of nulliparous women in Brazil determining resilience (Resilience Scale; RS) and stress (Perceived Stress Scale; PSS) at 28 weeks of gestation (± 1 week). Resilience and stress scores were compared according to sociodemographic characteristics related to maternal/perinatal outcomes and social vulnerability, defined as having low level of education, being adolescent, without a partner or ethnicity other than white.

**Results:**

We included 383 women who completed the RS and PSS instruments. Most women showed low resilience scores (median: 124.0; IQR 98–143). Women with a low resilience score (RS < 125) were more likely from the Northeast region, adolescents, other than whites, did not study or work, had a low level of education, low family income and received public antenatal care. Higher scores of perceived stress were shown in the Northeast, other than whites, at low levels of education, low annual family income and public antenatal care. Pregnant women with low resilience scores (*n* = 198) had higher perceived stress scores (median = 28) and at least one vulnerability criterion (*n* = 181; 91.4%).

**Conclusion:**

Our results reinforce the role of resilience in protecting women from vulnerability and perceived stress. It may prevent complications and build a positive experience during pregnancy.

**Supplementary Information:**

The online version contains supplementary material available at 10.1186/s12884-023-05692-5.

## Background

Pregnancy is a period of emotional challenges, arising from social and psychological factors and hormonal changes related to this phase [1]. This period of women´s life is full of changes and adaptations and a cognitive perception of uncontrollability and unpredictability, expressed in a physiological and behavioral response, is an ultimate definition of stress [2]. During pregnancy, stressors are related to both specific events and physiological adaptations of the maternal organism. Pregnancy symptoms include nausea, weight gain, insomnia, and emotional lability. Individual factors include unplanned pregnancy, changes in family dynamics, antenatal complications, or fear of developing complications [3, 4]. The socioeconomic context may also aggravate stressors for these pregnant women: low-income status, domestic violence, use of drugs and alcohol, lack of a family support network and other vulnerabilities [5]. Literature has demonstrated that three out of four pregnant women report some symptoms that indicate a level of stress [6]. Long-term exposure to stressors during pregnancy is associated with adverse maternal and perinatal outcomes, including premature rupture of membranes, preterm labor and small for gestational age fetuses [2, 6]. Studies have established an association between intrauterine stress and repercussions on cognitive and motor development and behavioral alterations in childhood [7]. A higher incidence of psychological disturbances occurs in women during pregnancy and postpartum [8]. These women require proper care and follow-up for adequate detection and intervention [9, 10].

Psychology has studied individual human reactions to adverse circumstances and/or stress factors, termed resilience [2, 11, 12]. This reaction is dependent of the intensity, frequency and level of stressors, and the response and coping mechanisms of the individual. The concept of resilience is the capacity to adapt to adversities in life. It is considered a subjective indication of this response, which encompasses internal strength, competence, and flexibility concepts, and may be inversely related to depression, perceived stress and anxiety [13, 14]. Some authors suggest that resilience may increase in adult life, probably deriving from a positive effect of overcoming limits and adversities during a lifetime [15, 16]. Resilience in women during pregnancy is still poorly studied.

Resilience should be assessed comparatively in a specific context and considering expected responses (e.g., same age group, social and cultural context, etc.) [17]. An individual in a context of vulnerability may be susceptible to higher exposure to risk factors such as health and economic constraints; vulnerability conditions can lead to different coping levels according to a particular context and individual characteristics. For instance, lower education, belonging to ethnic minority groups, higher work load, food insecurity and unhealthy habits are associated with barriers to health care, [5, 17, 18].

Women may fear the changes and physiological adaptations during pregnancy, childbirth, and the postpartum period [19, 20]. Resilience may help pregnant women cope with psychosocial problems, apart from pregnancy-specific concerns. Therefore, identifying less resilient groups in contexts of higher vulnerability may facilitate assisting women who are at higher risk and have less access to resources necessary to cope with some pregnancy-related process. This may contribute to the individual care of each pregnant woman and can support specific intervention strategies [21, 22]. It would be remarkably important for nulliparous women, who are facing maternity for the first time.

Although relevant for maternal and perinatal health, little is known about resilience during pregnancy and its determinants such as stress and maternal characteristics; similarly, studies addressing pregnancy outcomes related with lack of resilience are scarce. The current study aims to explore maternal resilience, perceived stress, and its association with vulnerability in a population of nulliparous pregnant women. Furthermore, the purpose is to evaluate the sociodemographic characteristics, health conditions and maternal and perinatal outcomes associated with different degrees of resilience and stress.

## Methods

This was a multicenter prospective cohort study. It was conducted in four referral obstetric care units in Brazil, within the Brazilian Network for Studies in Reproductive and Perinatal Health [23]*.* The primary objective of the MAES-I study (Maternal Actigraphy Exploratory Study – I) was to identify predictors of gestational complications, using data generated by wearable/mobile technology (wrist-worn sensors) to monitor sleep vigilance and physical activity. Methodological details and procedures related to the MAES-I study are described elsewhere [23]. Briefly, sample size calculation of the cohort was based on a 3 to 20% prevalence of major obstetric complications (e.g. preeclampsia, fetal growth restriction, gestational diabetes, bleeding complications). A theoretical population of more than 1 million pregnant women was considered, with an acceptable margin of error of 4%, and a 95% confidence interval, resulting in 384 women. The final sample was calculated at 400 pregnant women. This article follows the STROBE (*Strengthening the Reporting of Observational Studies in Epidemiology*) checklist for reporting a cohort study [24].

From March 2018 to March 2020, the four participating centers included nulliparous low-risk pregnant women, singleton pregnancy, gestational age confirmed between 19 and 21 weeks. Table S1 (Supplementary Material) shows that exclusion criteria were: history of ≥ 3 abortions, preexistent diabetes, stage II chronic hypertension or in use of medication, thyroid disease, kidney disease, HIV, hepatitis B or C, Systemic lupus erythematosus, antiphospholipid syndrome, sickle cell disease, suspicion of major fetal anomaly, antidepressant or anxiolytic use, any condition that limits the performance of physical activity, major uterine anomaly, cervical suture, knife cone biopsy, ruptured membranes, use of long-term steroids, low-dose aspirin, calcium (> 1 g/24 h), eicosapentaenoic acid (fish oil) > 2.7 g, vitamin C ≥ 1000 mg, vitamin E ≥ 400 UI, and heparin/LMW heparin, untreated thyroid disease. Data collection of epidemiological and clinical characteristics of the woman, pregnancy, childbirth, postpartum and newborn occurred during pregnancy at three antenatal visits (19–21, 27–29 and 37–39 weeks of gestation). In addition, a review of the medical records of mother and newborn was performed. During pregnancy, data collection included information on sociodemographic and anthropometric characteristics, maternal nutrition, lifetime habits, health history, gestational complications, resilience and stress.

Data collection on resilience and perceived stress occurred around 28 weeks (± 1 week). Pregnant women were interviewed in a private room in the antenatal care unit. Standardized and validated (self-administered) instruments were applied and records were transcribed to the *MedSciNet* web-based platform system.

Resilience was assessed by the Wagnild and Young Resilience Scale (1993), translated into Brazilian Portuguese, adapted transculturally and validated by Pesce et al. in 2005 [25, 26]. The original scale comprises 25 items, with a 7-point Likert scale, ranging from 1 (strongly disagree) to 7 (strongly agree); the total score ranges from 25 to 175. Scores over 145 indicate a high level of resilience, scores between 125 and 145 indicate a moderate level of resilience and scores under 125 indicate a low level of resilience [27].

Stress was evaluated with the perceived stress scale developed by Cohen et al. [28] and translated into Brazilian Portuguese and validated in 2007 by Luft et al. [29]. This scale has 14 items, 7 with a positive connotation and 7 with negative connotation, scoring from 0 to 4 (0 = never, 1 = almost never, 2 = sometimes, 3 = fairly often, 4 = very often). Questions with a positive connotation should be inversely added (0 = 4; 1 = 3; 2 = 2, 3 = 1 and 4 = 0), and negative questions should be added directly to their respective scoring values. The sum of all 14 items obtains the total scale score which does not have a cut-off for degrees of perceived stress. Scores may range from zero to 56. Higher perceived stress will score more points [28]. Questions from both instruments refer to the women’s perception from the last month.

Data of sociodemographic characteristics and pregnancy included Brazilian region (Southeast or Northeast, according to inclusion site); maternal age (categorized as ≤ 19 and > 19 years old); ethnicity/skin colour (self-reported and categorized as white and other than white), marital status (self-reported and categorized as with or without a partner), maternal occupation (self-reported and categorized as “Paid work or studying” or “Neither working nor studying”); schooling (self-reported and categorized as having had primary, secondary, college or higher education); monthly family income (self-reported local currency categorized as < 1,000, 1,001–2,000 and > 2,000 Brazilian *Reais* (BRL); estimated currency exchange rate at the time of the study was 1 US Dollar = 5 BRL); source of antenatal care, smoking, alcohol consumption, other drug use and history of any substance use. Data collection on maternal health conditions included urinary tract infection or any other infection in the first half of pregnancy, vaginal bleeding, hypertensive disorders (pre-eclampsia) and hospitalization in this period.

Vulnerability was defined by a theoretical-social concept based on five sociodemographic characteristics [30]: low level of education (less than 12 full years of schooling), adolescent (age 19 or younger), monthly family income < 1,000, without a partner during pregnancy (including single, divorced and widowed) or other than white ethnicity. In order to understand the impact of vulnerability, we thought to consider an analysis of its continuum as follows: no criterion of vulnerability, any criterium of vulnerability, exactly one criterion, exactly two criteria and three or more criteria.

Maternal and perinatal outcomes were onset of spontaneous labor, preterm birth, mode of birth (vaginal versus cesarean), time women stayed in hospital after childbirth (postnatal discharge), adequacy of birth weight, non-reassuring fetal status, fetal or neonatal death, neonatal intensive care unit (NICU) admission, low Apgar score, intubation at birth, preeclampsia, gestational hypertension, gestational diabetes, neonatal near-miss events, adverse perinatal outcome or any severe obstetric complication. Neonatal near miss was defined as having birthweight < 1750 g, 5^th^ minute Apgar < 7 or gestational age at birth < 33 weeks. Any adverse perinatal outcome (APO) was defined as having at least one of the following: NICU admission, intubation, hypoglycemia, 5th minute Apgar < 7, oxygen therapy or mechanical ventilation.

Resilience and perceived stress scores were analyzed by the distribution of measures of central tendency (the number used to represent the center or middle of a set of data values) and rate of resilience levels, according to the sociodemographic profile of the population. Chi-square test was used for percentage comparisons. Mann–Whitney U and Kruskal–Wallis tests were used to compare medians of two and three categorical variables. Bivariate analysis assessed the association between sociodemographic factors, maternal health and pregnancy with resilience and perceived stress. Correlation between resilience and perceived stress scores was assessed by Pearson´s correlation coefficient. Risk estimates for low resilience and high perceived stress were estimated according to degrees of vulnerability using risk ratios and 95% confidence intervals. Also, we calculated risk for pregnancy outcomes according to levels of resilience, using risk ratios and 95% confidence intervals. *P*-values < 0.05 were considered statistically significant. Bonferroni correction was applied to all analyses in order to test several hypotheses concurrently, while limiting type I error rate, brought on by inflation.

## Results

**Fig. 1:**
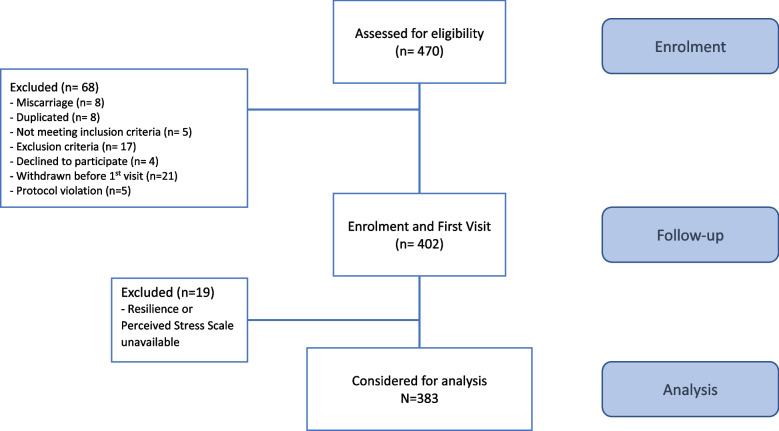
Flowchart of the MAES-I study – analysis of resilience and perceived stress during pregnancy

The MAES-I study identified 470 women as eligible to participate in the cohort and a total of 402 women were included (Fig. 1). For this analysis, 383 women had completed the resilience and perceived stress scales and answers were analyzed.

**Fig. 2:**
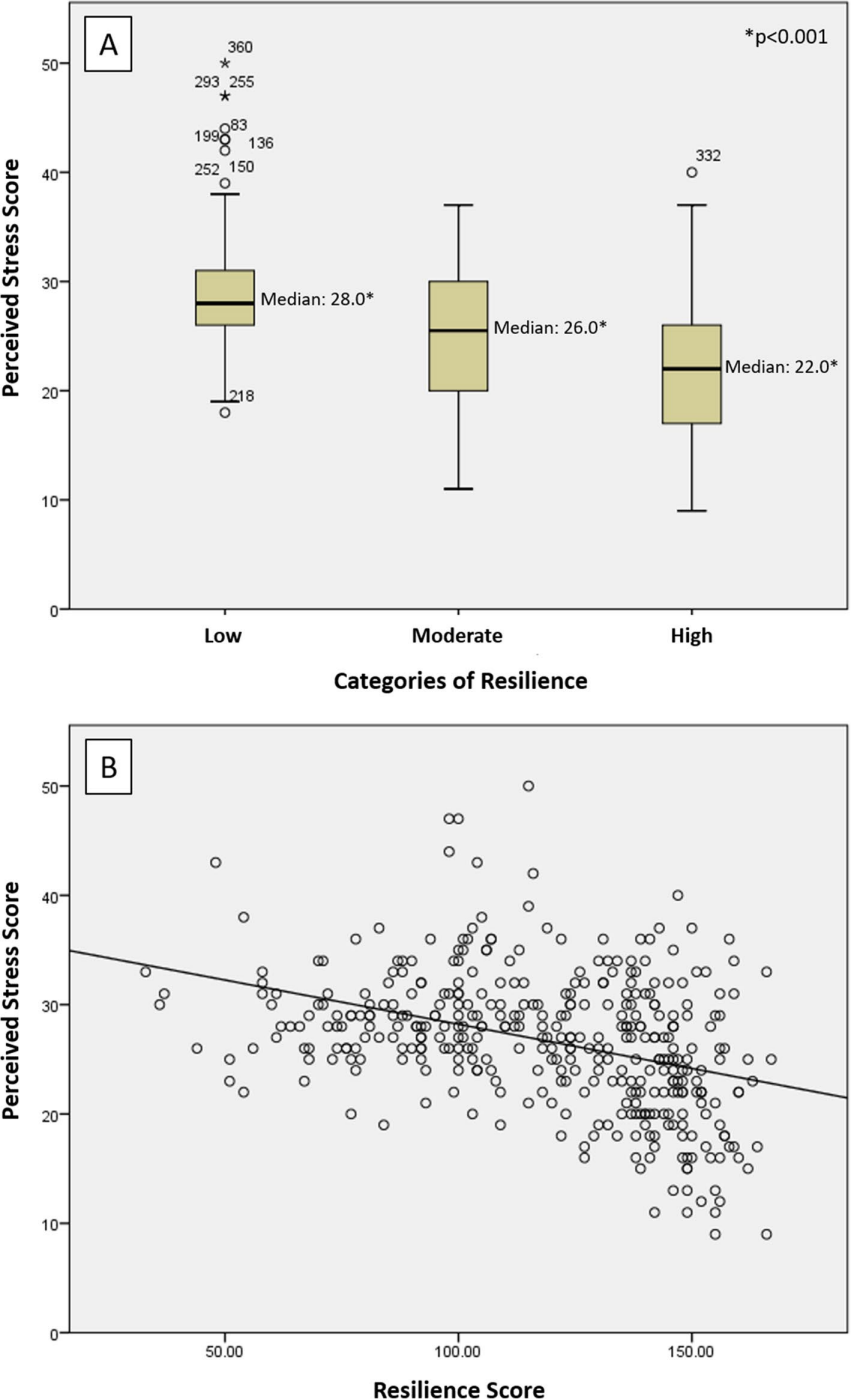
Association (**A**) and linear correlation (**B**) between Resilience and Perceived Stress scores among women from MAES-I study. Legend: **A** Distribution of maternal stress according to categories of resilience. Kruskal–Wallis test showed a significant difference of stress scores between groups (*p* < 0.001). **B** Pearson´s correlation coefficient of -0.379 (*p*-value < 0.001) shows that there was a significant linear correlation between resilience and perceived stress scores

Figure 2 addresses the association between perceived levels of stress and resilience. A moderately weak and negative linear correlation was observed between resilience and perceived stress scores (Pearson’s correlation coefficient -0.376, *p* < 0.001). The higher the level of resilience, the lower perceived stress.

Table 1 describes in detail the distribution of resilience and perceived stress. Mean and median resilience scores were 118.6 (Standard deviation: ± 29.4; range 33–167) and 124.0 (IQR: 98.0–143.0; 10^th^-90^th^ percentiles: 77.0- 152.0). Mean and median perceived stress scores were 26.7 (Standard deviation: ± 6.27; range 9.0–50.0) and 27.0 (IQR: 23.0–30.0; 10^th^-90^th^ percentiles: 18.0–34.0).

**Table 1:** Resilience and perceived stress score among participants of the MAES-I study

**Characteristics**	**Resilience score** *n* = 381	**Perceived Stress score** *n* = 381
Mean	118.5	26.7
Std Deviation	29.4	6.27
Minimum	33.0	9.0
Maximum	167.0	50.0
Percentile 5	66.1	16.0
Percentile 10	77.0	18.0
Percentile 25	98.0	23.0
Percentile 50 (median)	124.0	27.0
Percentile 75	143.0	30.0
Percentile 90	152.0	34.0
Percentile 95	156.0	36.0

*Std deviation* Standard deviation

When compared to highly resilient women, women with low resilience (*n* = 198) comprised of higher proportions of women living in the Northeastern region of Brazil (79.8%, *n* = 158/198), adolescents (28.8%; *n* = 57/198), other than whites (81.8%; *n* = 162/198), those who did not work or study (44.2%; *n* = 87/197), had lower schooling level (14.1%; *n* = 28/198), had monthly family income under 1,000 BRL (46.5%; *n* = 92/198) and received public antenatal care (95.5%; *n* = 189/198)(Table 2). At least one criterion of vulnerability was presented in 91.4% of the women with low resilience (*n* = 181/198). The majority of women with low resilience was non-smoking or had quit smoking when they knew they were pregnant (97.5%; *n* = 193/198), never used alcohol or stopped when they found out they were pregnant (94.4%; *n* = 187/198). Regarding any substance use (e.g. tobacco, alcohol, drugs or other drugs), 90.9% (*n* = 180/198) reported never using these substances during pregnancy.

**Table 2:** Distribution of resilience according to socio-demographic characteristics

	**Resilience**			
**Characteristics**	**Low** ***n*** ** = 198**	**Moderate** ***n*** ** = 100**	**High** ***n*** ** = 83**	***p*** **-value**
**Region**				** < 0.001**
Northeast	158 (79.8%)	27 (27.0%)	24 (28.9%)	
Southeast	40 (20.2%)	73 (73.0%)	59 (71.1%)	
**Maternal age**				**0.013**
≤ 19	57 (28.8%)	18 (18.0%)	12 (14.5%)	
> 19	141 (71.2%)	82 (82.0%)	71 (85.5%)	
**Ethnicity**				** < 0.001**
White	36 (18.2%)	51 (51.0%)	36 (43.4%)	
Other than white	162 (81.8%)	49 (49.0%)	47 (56.6%)	
**Marital status**				0.269
With partner	152 (76.8%)	74 (74.0%)	56 (67.5%)	
Without partner	46 (23.2%)	26 (26.0%)	27 (32.5%)	
**Maternal Occupation**^**a**^				**0.005**
Paid work or studying	110 (55.8%)	74 (74.0%)	57 (68.7%)	
Neither working nor studying	87 (44.2%)	26 (26.0%)	26 (31.3%)	
**Schooling**				**0.001**
Primary	28 (14.1%)	6 (6.0%)	1 (1.2%)	
Secondary	140 (70.7%)	67 (67.0%)	65 (78.4%)	
College or more	30 (15.2%)	27 (27.0%)	17 (20.5%)	
**Monthly Family Income (R$)**				** < 0.001**
0–1000	92 (46.5%)	18 (18.0%)	19 (22.9%)	
1001 to 2000	61 (30.8%)	29 (29.0%)	16 (19.3%)	
> 2000	45 (22.7%)	53 (53.0%)	48 (57.8%)	
**Source of antenatal care**				**0.011**
Public	189 (95.5%)	86 (86.0%)	78 (94.0%)	
Private/insurance/mixed	9 (4.5%)	14 (14.0%)	5 (6.0%)	
**Vulnerability**	181 (91.4%)	68 (68.0%)	64 (77.1%)	** < 0.001**
**Smoking**				**0.079**
Currently or during pregnancy	5 (2.5%)	4 (4.0%)	7 (8.4%)	
Never	193 (97.5%)	96 (96.0%)	76 (91.6%)	
**Alcohol drinking**				**0.033**
Currently or during pregnancy	11 (5.6%)	12 (12.0%)	12 (14.5%)	
Never	187 (94.4%)	88 (88.0%)	71 (85.5%)	
**Other drugs**				0.507
Currently or during pregnancy	3 (1.5%)	3 (3.0%)	3 (3.6%)	
Never	195 (98.5%)	97 (97.0%)	80 (96.4%)	
**History of use of any substance**				**0.005**
Currently or during pregnancy	18 (9.1%)	20 (20.0%)	18 (21.7%)	
Never	180 (90.9%)	80 (80.0%)	65 (78.3%)	
**Previous maternal conditions**	30 (15.2%)	29 (29.0%)	18 (21.7%)	**0.018**
**Urinary tract infection in the first half of pregnancy**	28 (14.1%)	15 (15.0%)	16 (19.3%)	0.548
**Vaginal bleeding in the first half of pregnancy**	31 (15.7%)	15 (15.0%)	14 (16.9%)	0.941
**Intercourse in the first half of pregnancy**	164 (82.8%)	89 (89.0%)	65 (78.3%)	0.144
**Occurrence of any infection in the first half of pregnancy**	62 (31.3%)	25 (25.0%)	33 (39.8%)	0.101
**Hospitalization in the first half of pregnancy**	3 (1.5%)	3 (3.0%)	0 (0%)	0.267

Missing information for a) 1

Maternal characteristics showing higher perceived stress scores were observed in the Northeastern region (median 28.0, *p*-value < 0.001), other than-whites (median 28.0, *p*-value < 0.019), with secondary level of education (median 28.0, *p*-value < 0.002) or lower (median 27.0, *p*-value < 0.002), family income between 1001,00 and 2000,00 BRL (median 29.0, *p*-value < 0.001) or less (median 28.0, *p*-value < 0.001), public antenatal care (median 27.0, *p*-value < 0.015), no history of drug use (median 27.0, *p*-value 0.014), no history of any substance use (median 27.0, *p*-value < 0.021) and low resilience scores (median 28.0, *p*-value < 0.001) (Table 3).

**Table 3:** Distribution of perceived stress according to socio-demographic characteristics

	**Perceived Stress Scale**					
**Characteristics**	**n**	**Median**	**IQR**	**Mean**	**SD**	***p*** **-value #**
**Region**						** < 0.001**
Northeast	209	28.0	25.0–30.5	27.9	5.2	
Southeast	172	25.0	20.0–30.0	25.2	7.0	
**Maternal age (years)**						0.112
≤ 19	87	28.0	25.0–31.0	27.4	5.3	
> 19	294	27.0	22.0–30.0	26.4	6.5	
**Ethnicity**						**0.011**
Other than white	260	28.0	24.0–31.0	27.3	5.9	
White	121	26.0	20.0–30.0	25.4	6.8	
**Marital status**						0.301
Without partner	99	28.0	23.0–31.0	27.4	6.4	
With partner	282	27.0	23.0–30.0	26.4	6.2	
**Maternal Occupation **^**a**^						0.381
Neither working nor studying	139	28.0	24.0–30.0	26.9	5.3	
Paid work or studying	241	27.0	22.0–31.0	26.5	6.7	
**Schooling (years)**						**0.002 ‡**
Primary	35	27.0	24.0–31.0	28.3	5.2	
Secondary	272	28.0	23.0–31.0	27.1	6.2	
College or more	74	25.0	20.0–28.2	24.4	6.3	
**Monthly Family Income (R$)**						** < 0.001 ‡**
0–1000	129	28.0	25.0–30.0	27.5	5.7	
1001 to 2000	106	29.0	25.0–31.0	28.0	5.3	
> 2000	146	25.0	20.0–29.2	25.0	6.9	
**Source of antenatal care**						**0.016**
Public	353	27.0	23.0–31.0	26.9	6.1	
Private/insurance/mixed	28	24.5	19.2–27.0	24.4	7.7	
**Vulnerability**						**0.001**
Yes	313	28	24.0–31.0	27.2	6.0	
No	68	25	19.0–30.0	24.5	6.7	
**Smoking**						0.951
Currently or during pregnancy	16	26.0	23.2–30.5	27.1	6.9	
Never	365	27.0	23.0–30.0	26.7	6.2	
**Alcohol drinking**						0.223
Currently or during pregnancy	35	26.0	21.0–29.0	26.4	7.7	
Never	346	27.0	23.0–31.0	26.7	6.1	
**Other drugs**						**0.014**
Currently or during pregnancy	9	22.0	18.5–26.0	22.2	4.2	
Never	372	27.0	23.0-31.0	26.8	6.2	
**History of use of any substance**						**0.024**
Yes	56	25.0	20.0–29.0	25.7	7.7	
No	325	27.0	23.0–31.0	26.8	5.9	
**Previous maternal conditions**						0.987
Yes	77	27.0	22.5–30.5	26.8	6.5	
No	304	27.0	23.0–30.0	26.7	6.2	
**Urinary tract infection in the first half of pregnancy**						0.075
Yes	59	28.0	25.0–32.0	28.1	7.0	
No	322	27.0	23.0–30.0	26.4	6.0	
**Vaginal bleeding in the first half of pregnancy**						0.160
Yes	60	28.0	25.0–31.0	27.9	6.9	
No	321	27.0	22.5–30.0	26.5	6.1	
**Intercourse in the first half of pregnancy**						0.727
Yes	318	27.0	23.0–30.0	26.6	6.1	
No	63	27.0	22.0–32.0	27.1	6.8	
**Occurrence of any infection in the first half of pregnancy**						0.451
Yes	120	27.0	23.2–31.0	27.1	6.5	
No	261	27.0	23.0–30.0	26.5	6.1	
**Hospitalization in the first half of pregnancy**						0.128
Yes	6	22.0	17.5–28.2	22.8	6.1	
No	375	27.0	23.0–30.0	26.7	6.2	
**Resilience**						** < 0.001 ‡**
Low	198	28.0	26.0–31.0	29.1	5.1	
Moderate	100	25.5	20.0–30.0	25.5	5.7	
High	83	22.0	17.0–26.0	22.4	6.8	

Missing information for a) 1. #Mann–Whitney U test for all comparisons, except for ‡ Kruskal–Wallis test. *IQR* interquartile range, *SD* standard deviation

The distribution of resilience and perceived stress in the studied population are presented in the supplementary material (Figures S1).

There was no significant difference between higher perceived stress scores and maternal age, marital status, maternal occupation, smoking, alcohol use, maternal comorbid conditions, baseline BMI at the first antenatal care visit, urinary tract infection or any other infection, vaginal bleeding, hospitalization and sexual intercourse in the first half of pregnancy.

Table 4 evaluated the estimated risks for low resilience and high perceived stress according to degrees of vulnerability. Women with at least one criterion of vulnerability had a higher risk of low resilience (RR 2.29; 95% CI 1.50–3.50), as well as those with only one criterion (RR 1.89; 95% CI 1.20–2.98), two criteria (RR 2.31; 95% CI 1.48–3.60) or three or more criteria (RR 2.77; 95% CI 1.80–4.27). Regarding the risk for perceived stress score above the 3rd quartile of the population sampled, only women with one criterion of vulnerability showed a statistically significantly increased risk (RR 1.96; 95%CI 1.07- 3.60). There was no significant association when only perceived stress ≥ 90^th^ percentile was analyzed.

**Table 4:** Risk estimates for low resilience and high perceived stress according to degrees of vulnerability (*n* = 383)

	**Vulnerability**								
	**None**	**Any**	**RR [95%CI]**	**Only one condition**	**RR [95%CI]**	**Two conditions**	**RR [95%CI]**	**Three or more conditions**	**RR [95%CI]**
**Resilience**									
Low	17 (25%)	181 (57.8%)	**2.31 [1.51–3.52]**	52 (47.3%)	**1.89 [1.20–2.98]**	59 (57.8%)	**2.31 [1.48–3.60]**	70 (69.3%)	**2.77 [1.80–4.27]**
Moderate/High	51 (75%)	132 (42.2%)	Ref	58 (52.7%)	Ref	43 (42.2%)	Ref	31 (30.7%)	Ref
**Perceived Stress Scale**									
≥ 3rd Quartile	11 (16.2%)	85 (27.0%)	1.66 [0.94–2.95]	35 (31.8%)	**1.96 [1.07–3.60]**	22 (21.6%)	1.33 [0.69–2.56]	26 (25.7%)	1.59 [0.84–3.00]
< 3rd Quartile	57 (83.8%)	230 (73.0%)	Ref	75 (68.2%)	Ref	80 (78.4%)	Ref	75 (74.3%)	Ref
≥ 90th centile	5 (7.4%)	42 (13.3%)	1.81 [0.74–4.41]	17 (15.5%)	2.10 [0.81–5.43]	11 (10.8%)	1.46 [0.53–4.03]	14 (13.9%)	1.88 [0.71–4.99]
< 90th centile	63 (92.6%)	273 (86.7%)	Ref	93 (84.5%)	Ref	91 (89.2%)	Ref	87 (86.1%)	Ref

Conditions considered as vulnerability criteria: low level of education (less than 12 complete years of schooling); adolescent (age 19 or younger); monthly family income < 1,000; without a partner during pregnancy (including single, divorced and widowed) or other than white

Maternal and perinatal outcomes of the sample population were analyzed according to levels of resilience (Table 5). Data on pregnancy outcomes from 372 women were available for analysis. There was no statistically significant difference between maternal and perinatal outcomes in women with low or moderate/high resilience. Outcomes were also analyzed in comparison to perceived stress scores in the sample. No statistically significant difference was observed between each outcome and perceived stress score (Table 6).

**Table 5:** Resilience and maternal and perinatal outcomes (*n* = 372)

	**Resilience**		
	**Low**	**Moderate/High**	**RR [95% CI]**
**Onset of Labour**			
Spontaneous	126 (66.0%)	115 (63.5%)	Ref
Induced/Elective C-section	65 (34.0%)	66 (36.5%)	0.94 [0.76–1.17]
**Preterm**			
pi-PTB	10 (5.2%)	4 (2.2%)	1.39 [0.98–1.96]
Spontaneous	10 (5.2%)	15 (8.2%)	0.77 [0.47–1.27]
No	171 (89.6%)	162 (89.6%)	Ref
**Mode of delivery**			
Vaginal	98 (51.3%)	99 (54.7%)	Ref
C-section	93 (48.7%)	82 (45.3%)	1.06 [0.87–1.30]
**Postpartum discharge **^**a**^			
1–3 days	152 (80.0%)	145 (81.5%)	Ref
> 3 days	38 (20.0%)	33 (18.5%)	0.95 [0.75–1.22]
**Non-reassuring fetal status **^**b**^	22 (19.6%)	23 (16.2%)	1.13 [0.81–1.59]
**Adequacy of birth weigh**			
SGA	25 (13.1%)	23 (12.7%)	0.99 [0.74–1.33]
AGA	155 (81.1%)	141 (77.9%)	Ref
LGA	11 (5.8%)	17 (9.4%)	0.75 [0.46–1.20]
**Fetal death**	1 (0.5%)	0 (0%)	-
**Neonatal death **^**c**^	0 (0%)	2 (1.1%)	-
**NICU admission**	12 (6.3%)	15 (8.3%)	0.85 [0.55–1.32]
**Low 5-min Apgar Score**	2 (1.0%)	2 (1.1%)	0.97 [0.36–2.60]
**Intubation at birth **^**c**^	2 (1.1%)	3 (1.7%)	0.77 [0.26–2.29]
**GDM **^**d**^	32 (22.1%)	29 (17.1%)	1.17 [0.89–1.55]
**Pre-eclampsia **^**e**^	14 (7.3%)	11 (6.1%)	1.10 [0.76–1.57]
**Any Great Obstetric Syndrome **^**f**^	58 (39.2%)	51 (29.7%)	1.24 [0.98–1.57]
**APO**	12 (6.3%)	16 (8.8%)	0.82 [0.53–1.27]
**Neonatal Near Miss**	7 (3.7%)	6 (3.3%)	1.05 [0.62–1.75]
**Maternal mortality**	1 (0.5%)	2 (1.1%)	0.64 [0.13–3.21]
**Total**	**191**	**183**	

Missing information for a) 4, b) 18, c) 1, d) 55, e) 48, f) 52 cases. *AGA* adequate for gestational age, *APO* adverse perinatal outcomes, *GDM* gestational diabetes mellitus, *LGA* large for gestational age, *NICU* neonatal intensive care unit, *pi-PTB* provider initiated Preterm Birth, *SGA* small for gestational age. APO was defined as having at least one of the following: NICU admission, intubation, hypoglycemia, 5^th^ minute Apgar < 7, oxygen therapy or mechanical ventilation

**Table 6:** Perceived stress and maternal and perinatal outcomes (*n* = 372)

	Stress					
	n	Median	IQR	Mean	SD	*p*-value#
Onset of Labour						0.164
Spontaneous	241	28.0	23.0–31.0	27.0	± 6.4	
Induced/Elective C-section	131	26.0	22.0–30.0	26.1	± 6.0	
Preterm						0.468‡
pi-PTB	14	30.5	20.5–29.0	27.9	± 6.8	
Spontaneous	25	28.0	23.5–29.0	26.5	± 5.0	
No	333	27.0	23.0–30.0	26.6	± 6.3	
Mode of birth						0.377
Vaginal	197	27.0	23.0–30.0	26.5	± 6.2	
C-section	175	27.0	23.0–31.0	26.9	± 6.3	
Postpartum discharge						0.918
1–3 days	297	27.0		26.7	± 6.5	
> 3 days	71	27.0		26.6	± 5.5	
Non-reassuring fetal status						0.364
Yes	45	27.0	22.0–32.0	27.3	± 6.6	
No	209	27.0	22.0–30.0	26.5	± 6.5	
Adequacy of birth weight						0.230‡
SGA	48	28.5	24.0–31.0	27.7	± 6.0	
AGA	296	27.0	22.0–30.0	26.6	± 6.3	
LGA	28	26.5	19.5–29.7	25.1	± 6.5	
Neonatal death						0.853
Yes	2	27.5	-	27.5	± 7.7	
No	369	27.0	23.0–30.0	26.7	± 6.3	
NICU admission						0.229
Yes	27	29.0	22.0–33.0	27.7	± 6.0	
No	345	27.0	23.0–30.0	26.6	± 6.3	
Low 5-min Apgar Score						0.670
Yes	4	24.5	20.5–32.2	25.7	± 6.2	
No	368	27.0	23.0–30.0	26.7	± 6.3	
Intubation at birth						0.654
Yes	5	25.0	20.0–31.5	25.6	± 6.2	
No	366	27.0	23.0–30.0	26.7	± 6.3	
GDM						0.571
Yes	61	26.0	23.0–29.5	26.3	± 5.4	
No	254	27.0	22.0–31.0	26.6	± 6.7	
Pre-eclampsia						0.715
Yes	25	26.0	25.0–30.5	26.4	± 5.4	
No	347	27.0	23.0–30.0	26.6	± 6.8	
Any major obstetric syndrome						0.944
Yes	109	27.0	23.0–30.0	26.6	± 5.3	
No	211	27.0	22.0–31.0	26.5	± 7.0	
APO						0.344
Yes	28	29.0	22.0–32.7	27.4	± 6.1	
No	344	27.0	23.0–30.0	26.6	± 6.3	
Neonatal Near Miss						0.338
Yes	13	29.0	23.5–32.5	28.0	± 5.5	
No	359	27.0	23.0–30.0	26.6	± 6.3	
Maternal mortality						0.905
Yes	3	27.0	-	25.6	± 9.0	
No	369	27.0	23.0–30.0	26.7	± 6.3	

^#^Mann–Whitney U test for all comparison, except for ‡ Kruskal–Wallis test. *AGA* adequate for gestational age, *APO* adverse perinatal outcomes, *GDM* gestational diabetes mellitus, *IQR* interquartile range, *LGA* large for gestational age, *NICU* neonatal intensive care unit, *pi-PTB* provider initiated Preterm Birth, *SGA* small for gestational age. APO was defined as having at least one of the following: NICU admission, intubation, hypoglycemia, 5^th^ minute Apgar < 7, oxygen therapy or mechanical ventilation. *SD* standard deviation

## Discussion

This is the first study to examine resilience, perceived stress and vulnerability in women with low-risk pregnancies. Low-resilient women had more social vulnerability-related characteristics, such as being from the Northeast, adolescent, other than white, low-educated, unemployed, from a low-income family and receiving public antenatal care. Such women often had higher perceived stress scores. There is a paucity of studies that apply the Wagnild & Young scale to assess resilience in pregnant women [25]. In general, resilience of a woman is measured indirectly, taking into consideration stress factors, depression, maturity, and self-esteem [31–33]. Salazar-Pousada et al. used a reduced version of the scale (version with 14 questions—RS14) in a case–control study [34] that evaluated depressive symptoms and resilience in pregnant adolescents [34]. The scale, however, was applied after birth in the postpartum period and not during pregnancy, which may have different implications on the interpretation of the context and significance of these results. Resilience is usually assessed in women experiencing a significant level of stress or health conditions during pregnancy. In a qualitative study, Kaye et al. evaluated resilience and vulnerability in 36 pregnant women admitted to hospital with severe complications (near-miss) [33]. Olajubu et al. assessed resilience (RS-14) and perceived stress (reduced version with 10 questions—PSS-10) in a population of 241 adolescents: 80.5% of the sample was categorized as having moderate levels of perceived stress related to pregnancy and 77.2% were classified as having low resilience; they also found an inverse relationship between perceived stress and resilience [35]. An American study by Johnson et al. [36] evaluated resilience using the 25-item Connor–Davidson Resilience Scale (CD-RISC 25) in a population of 30 pregnant women of a predominantly minority community, the majority of which were multiparous women with a mean antenatal resilience score of 82.0; similarly, Connor et al. found that the general population had a resilience score of 80.4 [36]. Connor’s study demonstrated the association between lower scores with a history of depression and antidepressant use, anxiety medication or insomnia and did not find an association between previous obstetric complications and substance abuse. We found that in our low-risk population of pregnant women, a higher proportion of women had low resilience scores (< 125, 51.7%); only 21.7% were classified as having high resilience (score > 145). These results raise some questions about 1) whether the cut-off points to classify degrees of resilience apply to obstetric populations; 2) the existence of particularities intrinsic to pregnancy that may be associated with higher rates of low resilience, such as fatigue, emotional distress, feeling overwhelmed by the sense of responsibility of taking-care.

In our study, women with low resilience and higher stress were more likely to have social vulnerability characteristics. Factors such as ethnicity, low level of education, low-income level, and lack of a partner during pregnancy, have already been explored in the literature in the context of possible effects on physical and mental health [37–39]. The most vulnerable women had worse gestational outcomes, either directly related to clinical complications or delays in identifying disease and health care provision [40]. It is believed that the presence of stress factors alone is not sufficient to promote alterations in physical or mental health, since it depends on individual perceptions of the stressor [41]. Furthermore, an individual can manage these factors.

Concerning the use of substances, our results were not consistent with data from the literature [42–44]. It is known that the use of drugs and/or alcohol may be considered as a mechanism to cope with stress [42, 43, 45]. Other studies using the perceived stress scale, have reported an association between alcohol use and high levels of stress [45]. Nevertheless, our data showed that in those with low resilience, there was a higher proportion of women that never smoked, drank, or used any type of substance. In contrast, the higher rates of perceived stress were not significantly related to smoking or alcohol use during pregnancy. Identification of the use of alcohol and drugs during pregnancy is challenging. While some voluntarily report their habit, others underestimate social use or hide for fear of stigmatization related to substance use during pregnancy. Therefore, self-reported data have less accuracy [44].

No significant associations between maternal and perinatal outcomes and resilience or perceived stress scores were found. Since it was a sample composed of low-risk nulliparous women, the frequency of expected adverse outcomes is usually low [46, 47]. Mgaya et al. published that multiparity was associated with higher maternal and perinatal risk compared to nulliparity [46]. The sample had a larger number of women under the age of 35, who generally have better perinatal outcomes [47, 48]. Also, the presence of a previous health condition, including diabetes, hypertension taking medication and thyroid disease, were exclusion criteria, which may have contributed to the low incidence of adverse effects in our sample. No classifications or value ranges exist for degrees of stress. This is another limitation that makes it difficult to interpret stress level across different groups. According to Cohen et al., statistical accuracy is reduced when the stress scale variable is categorized [28]. Therefore, two or five points higher in the stress scale is difficult to interpret in terms of clinical relevance.

Studies evaluating resilience in obstetric populations are scarce; further studies are necessary to better evaluate its relationship with maternal morbidity and pregnancy complications. It should also be considered that among outcomes in the MAES-I study, those related to mental health were not included [35, 49, 50]. Outcomes may also be affected by participation bias and the Hawthorne effect, since the participating women were known to be part of a study. These women were evaluated, interviewed, and examined during study visits by health care professionals (researchers). The examination comprised blood pressure measurement, urine strip test and diabetes monitoring, which may have improved antenatal care and prevented worse outcome [51].

Physiological adaptations of pregnancy, and typical pregnancy symptoms (e.g. nausea, lumbar pain, pelvic pain, constipation and insomnia) may affect a woman’s wellbeing. Childbirth and postpartum anxiety also contribute to a higher incidence of psychological disturbances during the gestational and puerperal periods [51, 52]. All these stressors may have different effects on maternal health [52, 53]. Individual evaluation of perceived stress, maternal resilience and identification of vulnerability criteria can increase the identification of individualized needs, giving the opportunity to provide broader individual health care, perceiving, preventing and treating adverse maternal and perinatal outcomes [52–54]. Antenatal care is a unique time to evaluate how a pregnant woman perceives stressors and withstand their effects, helping the establishment of bonds, promotion and stimulation of personal resources, and construction of a social support network that can provide a positive experience during pregnancy [54, 55].

In order to achieve comprehensive health care according to the pregnant women´s needs, it is essential to identify sociodemographic and psychosocial factors associated with increased stress or social vulnerability [56, 57]. In our study, vulnerability was associated with lower resilience and higher stress and it was considered as having one of the following conditions: low level of education (less than 12 complete years of schooling); adolescent (age 19 or younger); monthly family income < 1,000; without a partner during pregnancy (including single, divorced, and widowed) or other than white. The first point to discuss is that having a partner is not a guarantee of partnership. Having a partner disengagement during pregnancy or a partner who does not want the pregnancy, argues more, or is absent during childbirth is associated with higher levels of stress, anxiety, maternal depression, and other perinatal complications, including higher rates of fetal death [58, 59]. Therefore, it raises the importance of addressing support from partners and family´s individuals. Racial inequities are also an issue with worse antenatal, childbirth and postpartum care among other than white women [60]. Low maternal schooling is associated with increased maternal mortality, preterm birth, low birth weight and lower antenatal care attendance [61, 62]. Adolescence is associated with more adverse perinatal outcomes, hypertensive disorders of pregnancy, preterm birth and low birth weight [63].

A limitation of this study is that we did not address additional mental health aspects in the investigation, using standardized instruments [35, 49, 50]. Furthermore, similar to perceived stress, resilience was only assessed in pregnancy during one time period which might undermine the understanding of the resilience and stress throughout pregnancy. It should be highlighted that the dynamic nature of resilience and perceived stress refer to the “last month”. Nevertheless, there is a paucity of literature on this type of evaluation and further studies are required to identify the best time to evaluate and whether reassessment is necessary [35, 36]. Another limitation is that both scales were approved for research purposes only, preventing current clinical evaluation and contextualization of our data [26–28]. We envision, however, that these scales could be applied in an intervention study aiming to evaluate mental health in relation with pregnancy outcomes.

## Conclusions

This study reinforces the importance of a multidimensional approach to health care during pregnancy. Antenatal care is a window of opportunity to identify psychosocial predictors of vulnerability, perceiving contexts that provide scarce resources to overcome and reverse pregnancy stress factors. Therefore, access to resilience scores in pregnant women may be useful to develop individual and targeted coping strategies for support of women at higher risk. The field of mental health in pregnancy, focusing on the association of resilience, stress and vulnerability is still not fully understood. Further studies are necessary to reinforce the relevance of resilience and its role in preventing complications and construction of a positive experience in pregnancy.

## Supplementary Information

**Additional file 1: Table S1. **Inclusion and exclusion criteria of MaternalActigraphy Exploratory Study I (MAES-I). **Figure S1. **Distribution ofResilience scores among women from MAES-I study.

## Data Availability

The datasets used and/or analysed during the current study are available from the corresponding author on reasonable request.

## Abbreviations

APO: Adverse perinatal outcome
BMI: Body mass index
BRL: Brazilian Reais
CD-RISC 25: 25-Item Connor–Davidson Resilience Scale
CI: Confidence interval
IRB: Institutional Review Board
MAES-I: Maternal Actigraphy Exploratory Study – I
NICU: Neonatal intensive care unit
PSS: Perceived Stress Scale
PSS-10: 10-Item perceived stress scale
RR: Relative risk
RS: Resilience Scale
RS-14: 14-Item Resilience Scale
STROBE: Strengthening the Reporting of Observational Studies in Epidemiology
