# Supplementary material for: Measuring resilience and stress during pregnancy and its relation to vulnerability and pregnancy outcomes in a nulliparous cohort study
Source: BMC Pregnancy Childbirth. 2023 May 29;23:396. doi: 10.1186/s12884-023-05692-5 (PMC10226234; doi:10.1186/s12884-023-05692-5)
Supplement: Supplementary file 1 — Additional file 1: Table S1. Inclusion and exclusion criteria of MaternalActigraphy Exploratory Study I (MAES-I). Figure S1. Distribution ofResilience scores among women from MAES-I study. [file 12884_2023_5692_MOESM1_ESM.docx]

**Supplementary Material**

| **Table S1.** Inclusion and exclusion criteria of Maternal Actigraphy Exploratory Study I (MAES-I) |
| --- |
| **Inclusion criteria** |
| Singleton pregnancy |
| Nulliparous (who had never given birth before) |
| Between 19+0 and 21+0 weeks of gestation |
| **Exclusion criteria** |
| Unsure last menstrual period and unwilling to date the ultrasound. |
| ≥3 Miscarriages. |
| Major fetal anomaly/abnormal karyotype |
| Essential hypertension treated before pregnancy. |
| Moderate-severe hypertension at booking (≥160/100 mm Hg) or chronic hypertension using antihypertensive medication. |
| Prepregnancy diabetes. |
| Renal disease. |
| Systemic lupus erythematosus. |
| Antiphospholipid syndrome. |
| Sickle cell disease. |
| HIV or hepatitis B or hep C positive. |
| Any condition that limits the performance of physical activity. |
| Major uterine anomaly. |
| Cervical suture. |
| Knife cone biopsy. |
| Ruptured membranes. |
| Use of long-term steroids. |
| Use of low-dose aspirin. |
| Use of calcium (>1 g/24 hours). |
| Use of eicosapentaenoic acid (fish oil) >2.7 g. |
| Use of vitamin C ≥1000 mg and vitamin E ≥400 UI. |
| Use of heparin/LMW heparin. |
| Untreated thyroid disease. |
| Use of antidepressant and/or anxiolytic agents. |

**
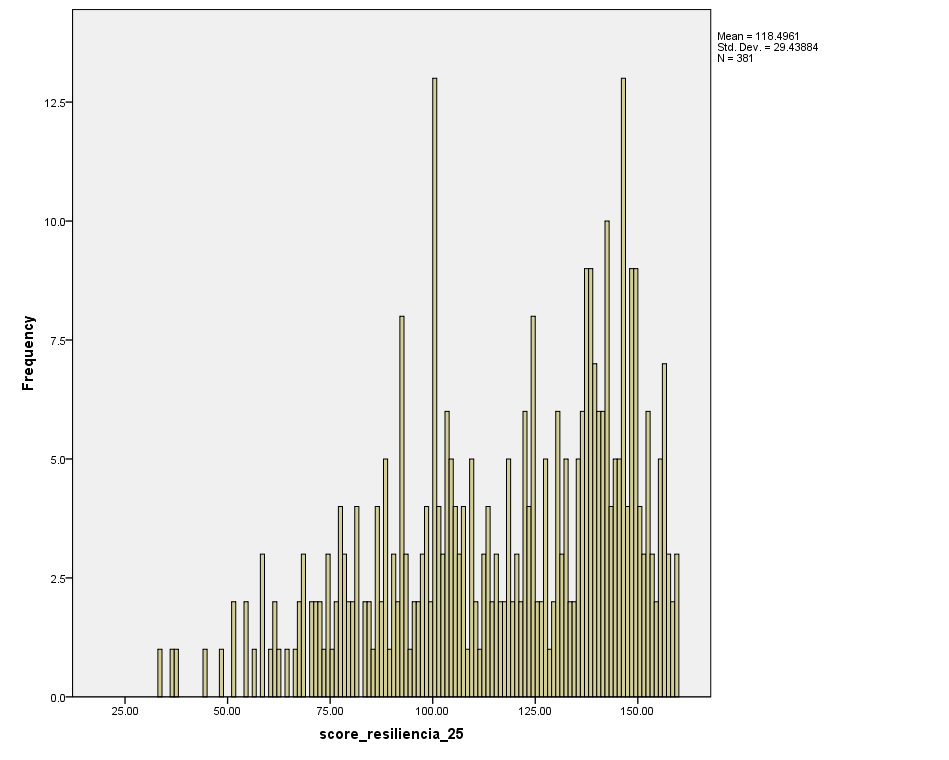
**

**Figure S1.** Distribution of Resilience scores among women from MAES-I study
